# Tailored Near‐Infrared Photoemission in Fluoride Perovskites through Activator Aggregation and Super‐Exchange between Divalent Manganese Ions

**DOI:** 10.1002/advs.201500089

**Published:** 2015-05-22

**Authors:** Enhai Song, Shi Ye, Tianhui Liu, Peipei Du, Rui Si, Xiping Jing, Sha Ding, Mingying Peng, Qinyuan Zhang, Lothar Wondraczek

**Affiliations:** ^1^ State Key Laboratory of Luminescent Materials and DevicesInstitute of Optical Communication MaterialsSouth China University of TechnologyGuangzhou510640China; ^2^ Beijing National Laboratory for Molecular SciencesState Key Laboratory of Rare Earth Materials Chemistry and ApplicationsCollege of Chemistry and Molecular EngineeringPeking UniversityBeijing100871China; ^3^ Shanghai Institute of Applied PhysicsChinese Academy SciencesShanghai Synchrotron Radiation FacilityShanghai201204China; ^4^ Otto Schott Institute of Materials ResearchUniversity of Jena07743JenaGermany

**Keywords:** Mn^2+^‐aggregation, near‐infrared emission, perovskites, photoluminescence, super‐exchange

## Abstract

Biomedical imaging and labeling through luminescence microscopy requires materials that are active in the near‐infrared spectral range, i.e., within the transparency window of biological tissue. For this purpose, tailoring of Mn^2+^–Mn^2+^ activator aggregation is demonstrated within the ABF_3_ fluoride perovskites. Such tailoring promotes distinct near‐infrared photoluminescence through antiferromagnetic super‐exchange across effective dimers. The crossover dopant concentrations for the occurrence of Mn^2+^ interaction within the first and second coordination shells comply well with experimental observations of concentration quenching of photoluminescence from isolated Mn^2+^ and from Mn^2+^–Mn^2+^ effective dimers, respectively. Tailoring of this procedure is achieved via adjusting the Mn–F–Mn angle and the Mn–F distance through substitution of the A^+^ and/or the B^2+^ species in the ABF_3_ compound. Computational simulation and X‐ray absorption spectroscopy are employed to confirm this. The principle is applied to produce pure anti‐Stokes near‐infrared emission within the spectral range of ≈760–830 nm from codoped ABF_3_:Yb^3+^,Mn^2+^ upon excitation with a 976 nm laser diode, challenging the classical viewpoint where Mn^2+^ is used only for visible photoluminescence: in the present case, intense and tunable near‐infrared emission is generated. This approach is highly promising for future applications in biomedical imaging and labeling.

## 1 Introduction

The transition metal ion Mn^2+^ is a vital component in the further development of lighting and display systems. There are a large variety of commercial phosphors such as Ca_5_(PO_4_)_3_Cl:Sb^3+^,Mn^2+^ (used in fluorescent lamps), Zn_2_SiO_4_:Mn^2+^, BaAl_12_O_19_:Mn^2+^ and BaMgAl_10_O_17_:Eu^2+^,Mn^2+^ (used in plasma display panels), or Eu^2+^/Mn^2+^‐codoped polycrystalline powders (used in high‐power white light‐emitting diodes).^1^, ^2^, ^3^, ^4^ Usually, in a solid matrix, the Mn^2+^ ion exhibits only visible (VIS) photo­luminescence, related to the relatively large energy gap (>17 000 cm^−1^) between the first excited level and the ground state of the 3d^5^ electronic configuration. In a situation that has been denoted as “normal photoluminescence,”^5^ when electrons are excited to energy levels above the emitting state, relaxation from these higher states occurs nonradiatively until the emitting state is reached. For Mn^2+^, normal photo­emission occurs from the state ^4^ *T*_1_(^4^ *G*) to the ground state ^6^ *A*_1_(^6^ *S*). This transition depends strongly on the crystal field of the ligands, what is most visible in the characteristic distinction between green luminescence that is typically associated with a tetrahedral coordination environment, ^IV^Mn^2+^ (i.e., lower ligand field strength), and orange or red luminescence from octahedral ^VI^Mn^2+^ (i.e., stronger ligand field).^6^, ^7^ Between those two species, the overall luminescence properties, in particular, emission color and quantum efficiency, can be tailored by controlling the precipitation of the Mn^2+^ activator.^8^, ^9^, ^10^

In addition to this “normal” VIS photoemission, a near‐infrared (NIR) emission band has recently been observed in association with Mn^2+^ doping at specifically elevated Mn^2+^ concentration.^11^, ^12^, ^13^ Such an additional emission band is undesirable for lighting and display applications that target the visible spectral range because it reduces the luminous efficacy of the phosphor by drawing on the excitation reservoir.^14^ The origin of such “anomalous” NIR emission from of Mn^2+^‐doped systems is presently not understood, which is mainly because only the one large energy gap between the ground state and the first excited state of Mn^2+^ has thus far been known to cause luminescence. The now‐observed NIR emission clearly cannot be attributed to the typically isolated Mn^2+^ ions; however, one possible explanation is given by the occurrence of Mn^2+^ ion aggregation. Interionic coupling between transition metal ions often leads to fascinating physical phenomena, such as ferromagnetism in Zn_1–*x*_Mn*_x_*Te^15^ and ZnO:Mn,^16^ multiferroism in BiFeO_3_ thin films,^17^ superconduction in Y/La–Ba/Sr–Cu–O ceramics,^18^, ^19^ and giant magnetoresistance in thin films of La_2/3_Ba_1/3_MnO*_x_* 20 and La_0.67_Ca_0.33_MnO*_x_*.^21^ These physical properties are primarily ascribed to the delocalization and interaction of the 3d electrons of transition metal ions. In analogy, coupling reactions between transition metal ions may result in novel luminescent centers with unusual luminescence behavior. This, in turn, can shed new light onto the nature of the underlying coupling reactions. Besides, luminescent materials that emit in the NIR (700–1100 nm) themselves are attractive for a variety of applications. Recently, the most vibrant field of interest in this context has been in the area of biomedical imaging, where cells and tissue exhibit weak autofluorescence and low transmission loss for optical signals just within the NIR.^22^, ^23^ It has hence been crucial to design (nano) materials that exhibit both emission and excitation of luminescence in the NIR region for in vitro and in vivo imaging applications.^24^, ^25^ For example, rare‐earth‐doped nanocrystals have attracted considerable interest in recent years as potential candidates for high‐resolution bioimaging because such nanocrystals may exhibit NIR upconversion (UC) emission upon excitation by a 976 nm laser diode (LD).^26^, ^27^, ^28^, ^29^ However, the accompanying intrinsically strong VIS emission that is additionally occurring in these rare‐earth‐doped materials limits the emission penetration depth in biological tissue because of the tissue's low transparency for wavelengths below 600 nm.^30^

In‐depth understanding and, consequently, knowledge‐based design of Mn^2+^‐activated materials that produce exclusively “anomalous” NIR emission, therefore, is of great significance from both theoretical and practical perspectives. Herein, we report the realization of anomalous and tailorable Stokes and anti‐Stokes single‐band NIR photoemission from Mn^2+^‐, and Mn^2+^/Yb^3+^‐doped perovskite‐type ABF_3_ via heavy doping with Mn^2+^ ions. Upon excitation with UV light or with an NIR LD, we observe anomalous NIR emission that can be adjusted between 760 and 830 nm. The well‐defined structure of ABF_3_ makes it an ideal and archetype model material for investigating the concentration‐dependent aggregation and luminescence behavior of Mn^2+^ ions. The underlying mechanism of anomalous NIR emission is consequently studied in this material by dynamic luminescence spectroscopy, analyses of the X‐ray absorption fine structure (XAFS), computational simulation through density functional theory (DFT), and through crystallographic analysis.

## 2 Results

**Figure 1:**
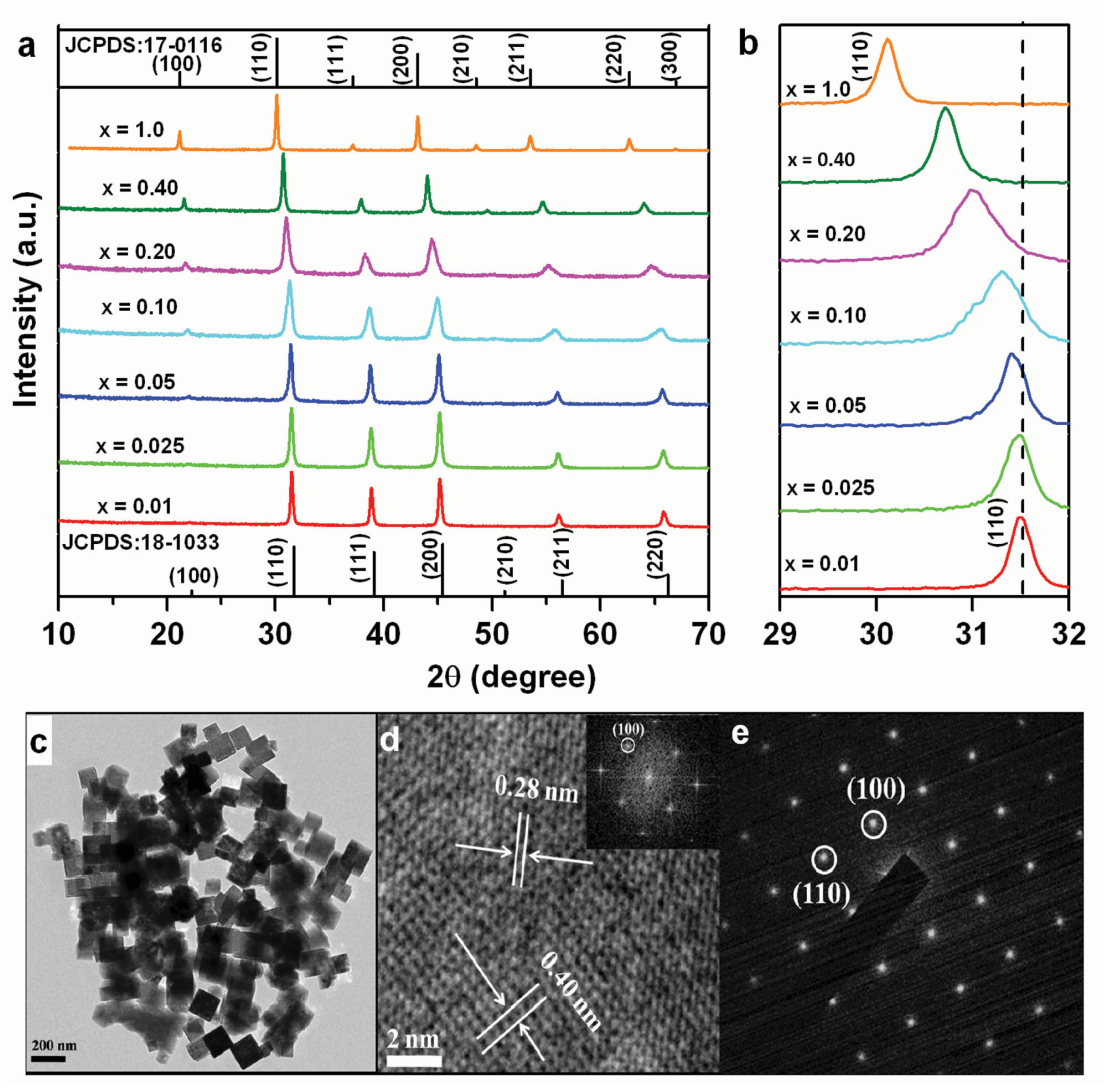
a) X‐ray diffraction patterns of KMg_(1–*x*)_F_3_:Mn^2+^ *_x_* for various Mn^2+^ doping levels (labels). Standard patterns of KMgF_3_ (JCPDS: 18‐1033) and KMnF_3_ (JCPDS: 17‐0116) are shown for reference; b) zoom at the angular region of 29–32°; c) typical TEM image for the sample with *x* = 0.1; d) high‐resolution TEM image of a single nanocrystal and corresponding Fourier transform (inset); and e) selected‐area electron diffraction pattern of a single nanocrystal.

The X‐ray diffraction (XRD) patterns of a series of KMgF_3_:Mn^2+^ samples and the corresponding JCPDS standard cards of KMgF_3_ and KMnF_3_ are shown in **Figure** **1**a. All of the observed diffraction peaks of the KMg_(1–*x*)_F_3_:Mn^2+^ *_x_* samples (with *x* ≤ 0.40) match well to those of JCPDS card no. 18‐1033 for cubic KMgF_3_. KMgF_3_ possesses a perovskite structure with space group *P*m–3m and lattice constants *a* = *b* = *c* = 3.98 Å. This structure is constructed from corner‐sharing MgF_6_ octahedra in which the Mg^2+^ ions are coordinated by six F^−^ ions, and the K^+^ ions are located in the tetrakaidecahedron cavity between the MgF_6_ octahedra (ICSD 94089). Increasing the Mn^2+^ content results in a gradual shift of all diffraction peaks to lower angles, i.e., to larger scattering units, because the partial replacement of the Mg^2+^ ions (*r* = 0.72 Å) by Mn^2+^ ions (*r* = 0.80 Å) leads to gradual lattice expansion (Figure 1b).^31^ After complete substitution, the diffraction peaks are in good agreement with those of the cubic perovskite phase KMnF_3_ (JCPDS card No. 17‐0116). A representative transmission electron microscopy (TEM) image is shown in Figure 1c for KMg_(1–*x*)_F_3_:Mn^2+^ *_x_* (with dopant fraction *x* = 0.10). It clearly reflects the quasicubic shape of the as‐synthesized nanocrystals, with an average size of ≈200 nm. Figure 1d shows a typical high‐resolution TEM image of a single such crystal. The spacing of the lattice planes is 0.40 and 0.28 nm, which is consistent with the (100) and (110) lattice planes (*d*_100_ = 0.398 nm and *d*_110_ = 0.282 nm), respectively, of cubic KMgF_3_ (JCPDS card No. 18‐1033). The slight increase in the observed d values is assumedly caused by the partial substitution of Mg^2+^ ions by Mn^2+^ ions. The Fourier transform (FT) of the high‐resolution TEM image (inset in Figure 1d) and the regular selected area electron diffraction patterns (Figure 1e) confirm that the synthesized particles are KMgF_3_ single crystals.

**Figure 2:**
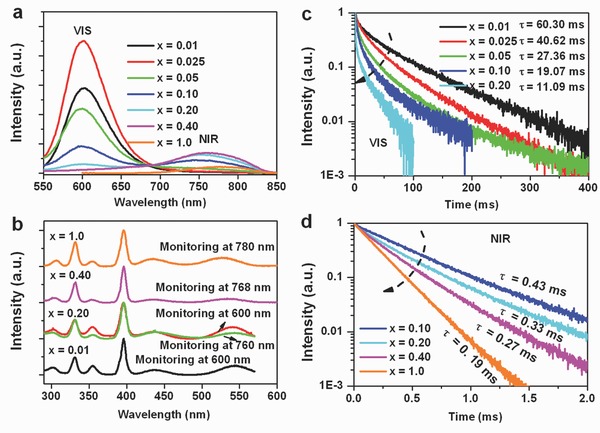
a) Room temperature emission spectra upon excitation with 396 nm UV light; b) excitation spectra, monitoring VIS and NIR emission bands; c) VIS luminescence decay curves; and d) NIR luminescence decay curves.

The emission spectra of KMg_(1–*x*)_F_3_:Mn^2+^ *_x_* with varying Mn^2+^ content (0.01 ≤ *x* ≤ 1.0) are shown in **Figure** **2**a for an excitation wavelength of 396 nm. For *x* < 0.10, the emission spectra consist of a single VIS emission band that is centered at ≈600 nm. This corresponds to normal photoluminescence due to the ^4^ *T*_1_(*G*)→^6^ *A*_1_(*S*) transition in Mn^2+^. However, when the Mn^2+^ concentration reaches *x* = 0.10, the anomalous NIR emission band appears at ≈760 nm, together with the VIS emission. At the same time, the VIS emission band undergoes a slight blueshift, which we attribute to the gradually reducing ligand field strength. The NIR emission band, on the other side, redshifts with further increasing Mn^2+^ concentration, i.e., 0.10 ≤ *x* ≤ 1.0. This, in turn, indicates that the NIR and VIS emission procedures are characteristically different and do not share a single origin. Band maxima are observed for *x* = 0.025 (VIS) and *x* = 0.40 (NIR), respectively. In particular, a doping concentration of Mn^2+^ of *x* ≥ 0.40 produces pure NIR emission (with a band maximum at 768 nm), implying that the VIS band is quenched‐out at this dopant concentration. This is another evidence for the different origins of the VIS and NIR bands. Second, this indicates that at sufficiently high dopant concentration, the energy that is absorbed on the Mn^2+^ ions is fully converted into NIR radiation. The excitation spectra and luminescence decay curves of the two emission bands are reported in Figure 2. Both bands exhibit similar excitation spectra (Figure 2b). Moreover, there is only little change in the positions of the excitation bands with increasing Mn^2+^ content, indicating that both emission bands are directly associated with Mn^2+^ content. The excitation peaks at 302, 331, 354, 396, 438, and 541 nm are due to electronic transitions of Mn^2+^ from the ground state ^6^ *A*_1_(*S*) to the excited states ^4^ *T*_1_(*P*), ^4^ *E*(*D*), ^4^ *T*_2_(*D*), [^4^ *A*_1_(*G*), ^4^ *E*(*G*)], ^4^ *T*_2_(*G*), and ^4^ *T*_1_ *(G*), respectively. In Figure 2c, the luminescence decay curves of the VIS emission are shown for *x* ≤ 0.10. The VIS emission dynamics clearly deviate from a single‐exponential decay function, what points to the participation of multiple processes in the VIS radiative relaxation. The effective lifetime of the VIS emission band decreases monotonically from ≈60.3 to ≈11.1 ms as the Mn^2+^ concentration increases from *x* = 0.01 to *x* = 0.20. In contrast, the NIR emission exhibits nearly single exponential decay behavior, as shown in Figure 2d. Here, the effective decay times are approximately 0.43, 0.33, 0.27, and 0.19 ms for Mn^2+^ doping levels of *x* = 0.10, 0.20, 0.40, and 1.0, respectively.

Since the divalent manganese ion (Mn^2+^) has a 3d^5^ electronic configuration with only one large gap between all the adjacent energy levels, luminescent materials activated by Mn^2+^ ions exhibit only one emission peak at a particular occupied site.^6^ As previously mentioned, the 3d electrons of Mn^2+^ ions are not fully localized. In particular, when Mn^2+^ ions are sufficiently close to each other, i.e., at lateral separations of only several angstroms,^32^, ^33^ Mn^2+^‐Mn^2+^ super‐exchange interactions occur alongside bridges that connect two Mn^2+^ cations via another anion, here F^−^. Then, the Mn^2+^‐Mn^2+^ pair may be treated as an effective dimer, which possesses a different emission band as compared to that of the isolated Mn^2+^ ion: ^6^ *A*_1_(^6^ *S*)^6^ *A*_1_(^6^ *S*) and ^6^ *A*_1_^4^ *T*_1_(^4^ *G*) have been suggested as the ground state and the first excited state (emitting state), respectively, of the effective dimer.^32^ In the following, we intend to exploit this behavior as a novel approach for tailoring the emission wavelength of Mn^2+^‐based phosphors, whereby the Mn^2+^ doping concentration is varied to control the distance between the Mn^2+^ ions in the crystal lattice. That is, aggregation of Mn^2+^ ions in a suitable host lattice produces a new emission band, ${}^{6}A_{1}{{(}^{6}S)}^{4}T_{1}{(}^{4}G)\to^{6}A_{1}{{(}^{6}S)}^{6}A_{1}{(}^{6}S)$. Increasing the Mn^2+^ concentration allows for the formation of effective Mn^2+^‐Mn^2+^ dimers via super‐exchange in KMgF_3_:Mn^2+^. The excitation spectra and luminescence decay lifetime data shown in Figure 2 indicate that the NIR emission in KMgF_3_:Mn^2+^ (*x* ≤ 0.4) and KMnF_3_ is of the same nature and origin.

**Figure 3:**
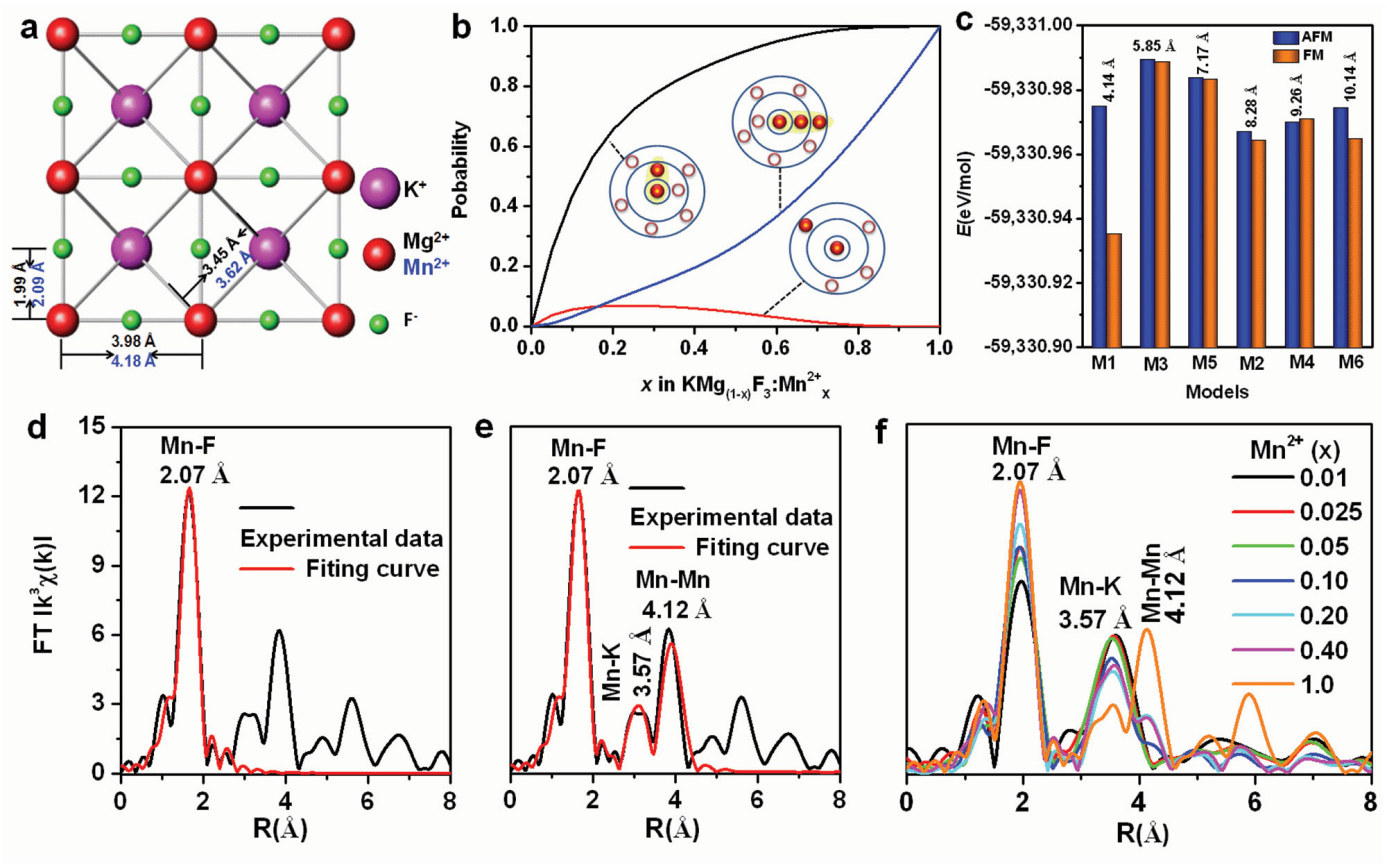
a) Crystal structure of KMg(Mn)F_3_; b) probability for the occurrence of specific Mn^2+^‐Mn^2+^ coordination environments (see text for details); c) energy of formation (*E*) for six possible situations in a 2 × 2 × 4 supercell of KMgF_3_:Mn^2+^ with two Mg^2+^ ions substituted by two AFM or FM‐coupled Mn^2+^ ions (denoted as M1, M2, M3, M4, M5, and M6 with different shortest Mn–Mn distances); d) FT *k^3^*‐weighted *χ*(*k*)‐function of EXAFS spectrum for Mn^2+^ in KMnF_3_ with Mn–F fitting; e) FT *k^3^*‐weighted *χ*(*k*)‐function of EXAFS spectrum for KMnF_3_ with Mn–F, Mn–K, and Mn–Mn fitting; and f) *k^3^*‐weighted *χ*(*k*)‐function of EXAFS spectra for KMgF_3_:Mn^2+^ at various Mn^2+^ doping levels.

To validate our hypothesis, we investigated the possibility of Mn^2+^ ion aggregation in KMgF_3_:Mn^2+^ by using both simulations and experiments. **Figure** **3**a shows that the nearest Mg^2+^–Mg^2+^ distance in cubic KMgF_3_ is 3.98 Å, whereas the nearest Mg^2+^—F^−^ and Mg^2+^—K^+^ distances are approximately 1.99 and 3.45 Å (ICSD 94089), respectively. For Mn^2+^‐doped KMgF_3_, the Mn^2+^ ions substitute on Mg^2+^ sites that have the same charge and approximately the same ionic size (Mg^2+^: *r* = 0.72 Å; Mn^2+^: *r* = 0.80 Å). Therefore, at a sufficiently high doping concentration, Mn^2+^(Mg^2+^)‐Mn^2+^(Mg^2+^) dimers are likely to form over one F^−^ anion bridge in this system. A simple statistical consideration^34^ of the probability for the existence of {—Mn—F—Mn—}, {—Mn—F—Mg—F—Mn—}, and {—Mn—F—Mn—F—Mn—} entities, respectively, is done on the basis of corner‐sharing MnF_6_ and/or MgF_6_ octahedra where each octahedron is connected to six other octahedra. It yields a distribution as shown in Figure 3b. Here, the {—Mn—F—Mn—} entity is an effective dimer, the {—Mn—F—Mn—F—Mn—} entity represents two interacting effective dimers, and {—Mn—F—Mg—F—Mn—} stands for isolated Mn^2+^ species where no other Mn^2+^ ion is found in the first coordination shell. It can be seen from Figure 3b that the concentration of the latter species decreases continuously when the concentration of Mn^2+^ exceeds *x* = 0.25. This coincides with the observation of VIS luminescence, which undergoes notable concentration quenching from this concentration upward. The probability for the formation of neighboring effective clusters, {—Mn—F—Mn—F—Mn—}, is low at first, but increases strongly for *x* > 0.40. Here, as well, concentration quenching is observed for NIR luminescence at *x* > 0.40.

To further elaborate this hypothesis, we perform first‐principle calculations on the lattice geometry following substitution by using DFT for a 2 × 2 × 4 supercell of KMgF_3_:Mn^2+^ with two Mg^2+^ ions substituted by two Mn^2+^ ions. The cell parameters of the initial supercell are listed in Table S1 (Supporting Information). There are six possible substitution models as illustrated in Figure S1 (Supporting Information). The computational results are listed in Tables S2–S4 (Supporting Information). Since the optimized cell parameters and the crystal structures of all models do not vary significantly between each other, the derived data appear convincing. Here, the energy of formation *E* is the energetic difference between the crystalline unit cell and the isolated constituting atoms.^35^ As seen in Figure 3c, the M1 model with shortest Mn^2+^‐Mn^2+^ distance and antiferromagnetic (AFM) interaction has a lower formation energy than the model M1 with ferromagnetic (FM) interaction. Other situations with Mn^2+^‐Mn^2+^ distance larger than 5 Å exhibit little energetic difference for the two types of interactions, which suggests that AFM interaction lowers the energy for the model with neighboring Mn^2+^‐Mn^2+^, and high Mn^2+^‐Mn^2+^ distance leads to the absence of any AFM or FM interaction. The situation denoted as M1 in which AFM occurs as a result of Mn^2+^ aggregation with a Mn^2+^‐Mn^2+^ distance of about 4.14 Å possesses, in this context, a relatively low energy of formation. We hence assume that this is the most probable situation which occurs when a certain Mn^2+^ doping concentration is reached. This is in line with the experimental observation of AFM in KMnF_3_ and KMgF_3_:Mn^2+^.^36^, ^37^

**Figure 4:**
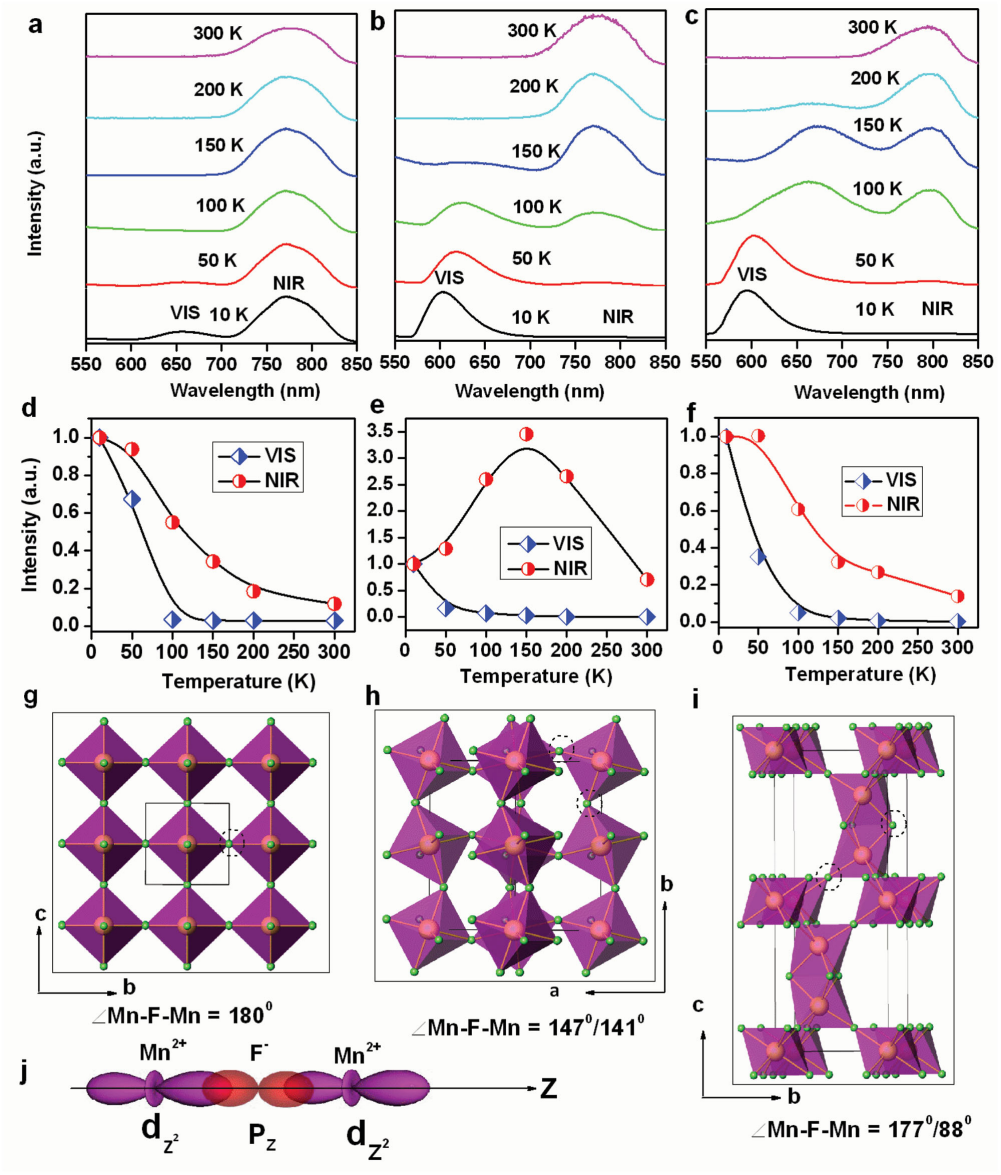
PL spectra of a) KMnF_3_; b) NaMnF_3_ and c) CsMnF_3_ at various temperatures ranging from 10 to 300 K; d–f) show variations of temperature‐dependent emission intensities of VIS and NIR emissions for KMnF_3_, NaMnF_3_, and CsMnF_3_, respectively; g–i) crystal structures of KMnF_3_, NaMnF_3_, and CsMnF_3_, respectively (for clarity, the alkali cations are omitted in the structures and only the MnF_6_ octahedra are shown); and j) schematic representation of the most important sigma overlaps between the d orbitals of Mn^2+^ and the p orbital of ligand F^−^ with a bridging angle of 180°.

XAFS, comprising X‐ray absorption near‐edge spectroscopy (XANES) and extended X‐ray absorption fine structure (EXAFS), was used to provide complementary information on the electronic and local geometric configuration in the present KMgF_3_:Mn^2+^ compound. These experiments were done on the beamline BL14W1 at the Shanghai Synchrotron Radiation Facility (SSRF). Mn K‐edge (XANES) spectra were measured for the KMgF_3_:Mn^2+^ samples as shown in Figure S2 (Supporting Information). The Fourier transform (FT) *k* ^3^‐weighted *χ*(*k*)‐functions of the KMnF_3_–EXAFS spectrum with Mn–F, Mn–K, and Mn–Mn shells fitting are shown in Figure 3d–e. For the Mn–F fit to the KMnF_3_ sample, a coordination number (*N*) of 6 for the Mn^2+^ ions is obtained, clearly confirming the assumption of octahedral configuration. Fits for the Mn–F, Mn–K, and Mn–Mn shells are used to determine the respective interionic distances, 2.07, 3.57, and 4.12 Å, respectively. These values are in good agreement with the ones of cubic perovskite KMnF_3_ (Figure 3a). The EXAFS spectrum of KMnF_3_ was used as a reference to compare the EXAFS spectra of samples with various Mn^2+^ contents, i.e., KMg_(1–*x*)_F_3_:Mn^2+^ *_x_* for 0.01 ≤ *x* ≤ 0.40 (see Figure 3f). For a Mn^2+^‐doping concentration below *x* = 0.10, only the Mn–F and Mn–K peaks can be observed in the fits of the EXAFS spectra, indicating that the still isolated Mn^2+^ ions are randomly and homogeneously distributed in the host lattice at such low dopant concentrations. However, when the Mn^2+^ doping concentration rises to *x* = 0.10, also the peak attributed to Mn–Mn appears in the spectra, which is taken as experimental evidence for Mn^2+^ ion aggregation in KMgF_3_:Mn^2+^. This observation appears to be in good agreement with both the Stokes emission behavior of KMgF_3_:Mn^2+^ for increasing Mn^2+^ content, and with the DFT results. Therefore, we conclude at this point that the Mn^2+^‐related NIR emission in perovskite KMgF_3_:Mn^2+^ originates from Mn^2+^‐Mn^2+^ effective dimers that form by statistical aggregation of Mn^2+^ ions in the form of {—Mn—F—Mn—} bridges. It should be noted, though, that aggregation would not by itself lead to distinct luminescence^38^, ^39^ because there has to be some kind of interaction between the activators. Such interaction reactions between transition metal ions are generally dependent on orbital and ligand geometry.^40^ In order to obtain a deeper understanding of this, we recorded the temperature‐dependent emission spectra for Mn^2+^‐doped KMnF_3_, NaMnF_3_, and CsMnF_3_, in which different alkali ions are used to provide different Mn^2+^—Mn^2+^ linkage geometry. The corresponding XRD patterns reveal a pure perovskite structure for all three as‐synthesized materials (Figure S3, Supporting Information). **Figure** **4**a shows the emission spectra of KMnF_3_ at different temperatures within 10–300 K under 396 nm light excitation. The 10 K emission spectrum consists of a broad VIS band centered at 656 nm, and a NIR band at approximately 780 nm, which is quite different from the room temperature emission spectrum. For one, the NIR emission is much stronger than the VIS emission at 10 K. Monitoring the emission wavelengths at 656 and 780 nm reveals similar excitation spectra for both bands (Figure S4a, Supporting Information), indicating that both emission bands are associated with Mn^2+^ ions. The VIS emission band is ascribed to the ^4^ *T*_1_(*G*)→^6^ *A*_1_(*S*) transition in Mn^2+^ ions, as commonly reported in the literature,^11^, ^41^, ^42^ whereas the NIR emission band, as already argued, is assigned to the presence of effective Mn^2+^‐Mn^2+^ dimers. Increasing the temperature leads to a significant decrease in the intensity of both emission bands because of the more and more prominent interference of nonradiative relaxation (Figure 4d). As the temperature is increased to 100 K, the VIS emission is almost fully quenched, whereas the NIR emission can be observed even at room temperature. These results clearly show that thermal quenching has a much more significant effect on the normal Mn^2+^ luminescence than it has on the proposed Mn^2+^‐Mn^2+^ dimer emission. NaMnF_3_ and CsMnF_3_ are studied to verify the aggregation‐induced geometry‐dependent coupling of Mn^2+^ ions (see Figure 4b,c). At room temperature, the emission spectra of the two compounds exhibit solely NIR emission with band maxima at approximately 772 and 795 nm, respectively, very similar to KMnF_3_. The spectra for orthorhombic NaMnF_3_ and hexagonal CsMnF_3_ at 10 K show dominance of the VIS emission band at ≈600 nm. Again, the excitation spectrum of this band is similar to that of the NIR emission at room temperature (Figure S4b,c, Supporting Information). For NaMnF_3_, the VIS emission intensity sharply decreases as the temperature increases, whereas the NIR emission increases and reaches a maximum at 150 K before decreasing monotonically to almost its original level (Figure 4e). When the temperature reaches 200 K, the VIS emission is completely quenched. The low temperature emission spectra of hexagonal CsMnF_3_ are similar to those of orthorhombic NaMnF_3_. Both the VIS and NIR emission intensities gradually decline with temperature (Figure 4f), similarly as in KMnF_3_ (Figure 4d). Here, VIS emission is completely quenched at 300 K. These results confirm that the nature of the NIR and VIS emission bands is the same in all three different perovskite compounds. The differences in the temperature‐dependent quenching are attributed to differences in the Mn^2+^‐Mn^2+^ linkage configurations.

In Figure 4g, we illustrate that the MnF_6_ octahedra in the cubic perovskite KMnF_3_ are connected through a common F atom at a Mn—F—Mn angle of 180°. Orthorhombic perovskite NaMnF_3_ is a somewhat distorted version of the ideal perovskite structure in which the MnF_6_ octahedra are tilted, and the connecting angle of Mn—F—Mn is 147°/141° because of the small radius of the Na^+^ cation, as shown in Figure 4h. For hexagonal CsMnF_3_ (Figure 4i), the MnF_6_ octahedra share a corner and a face with a connecting angle of 177°/88°. It has been reported that the exchange interaction between two coupling ions is primarily governed by the overlap of the orbital wavefunctions for linear configuration,^40^, ^43^ see Figure 4j. Thus, enhanced coupling between Mn^2+^ ions is expected in KMnF_3_ and CsMnF_3_ because these compounds have nearly linear Mn—F—Mn configuration. For the tilted linkage in NaMnF_3_, only reduced coupling is expected. As a consequence, VIS emission is less prone to concentration quenching. Thermal quenching effect is responsible for the variation in the NIR emission intensity in KMnF_3_ and CsMnF_3_ with varying temperature. On the other side, the significant increase in the NIR emission of NaMnF_3_ with temperature (Figure 4e) suggests that interionic coupling is enhanced with temperature in the tilted NaMnF_3_ structure, associated with the less‐and‐less strong localization of the d electrons of the Mn^2+^ ion. This hypothesis is corroborated by the gradual shift in the VIS emission peaks to longer wavelength with increasing temperature. The decrease of the NIR emission in NaMnF_3_ above 150 K is then attributed to the competing effect of thermal quenching.

**Figure 5:**
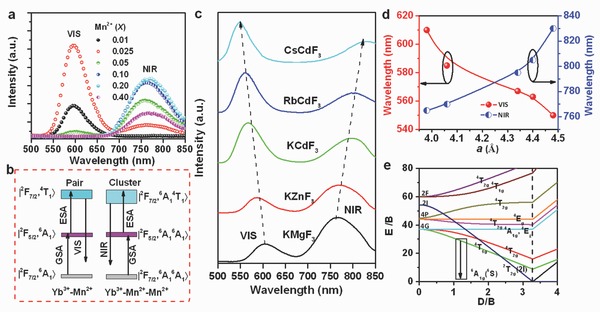
a) Room‐temperature upconversion emission spectra of KMg_(1–*x*–*y*)_F_3_:Yb^3+^ *_y_*/Mn^2+^ *_x_* (0.01 ≤ *x* ≤ 0.40; *y* = 0.005); all samples were excited with a 976 nm LD at a power density of 10 W cm^−2^; b) proposed two‐photon upconversion mechanism for KMgF_3_:Yb^3+^,Mn^2+^; c) room temperature upconversion emission spectra of AB_(1–*x*–*y*)_F_3_:Yb^3+^ *_y_*/Mn^2+^ *_x_* (*x* = 0.20; *y* = 0.005; A = K, Rb, Cs; B = Mg, Zn, Cd); here, too, all samples were excited with a 976 nm LD at a power density 10 W cm^−2^; d) emission peak positions of VIS and NIR emissions from AB_(1–*x*–*y*)_F_3_:Yb^3+^ *_y_*/Mn^2+^ *_x_* (*x* = 0.20; *y* = 0.005) as a function of the lattice constant *a* for perovskite KMgF_3_, KZnF_3_, KCdF_3_, RbCdF_3_, and CsCdF_3_; and e) Tanabe–Sugano energy diagram of a 3d^5^ system in an octahedral crystal field.

To further investigate Mn^2+^ UC emission, we considered the introduction of Yb^3+^ as a codopant into the KMgF_3_:Mn^2+^ lattice. **Figure** **5**a shows the UC emission spectra of KMg_(1–*x*–*y*)_F_3_:Yb^3+^ *_y_*/Mn^2+^ *_x_* (0.01 ≤ *x* ≤ 0.40; *y* = 0.005) after excitation with a 976 nm LD. For the sample with *x* = 0.01, the emission spectrum consists of a single VIS emission band centered at 595 nm, which corresponds to the |F27/2,4T1g(G)→|F27/2,6A1g(S)〉 transition of the Yb^3+^—Mn^2+^ pair.^44^ Increasing the Mn^2+^ doping concentration to *x* = 0.025 results in the appearance of an anomalous NIR emission band at ≈760 nm, in addition to the VIS emission band. A sole NIR emission band is obtained at doping concentrations *x* ≥ 0.10. The NIR UC emission band is located within the transmission window of biological tissue. Additionally, we find a high thermal stability of the UC NIR emission band (Figure S5, Supporting Information). We hence conclude that such materials could have potential applications in high‐resolution and deep‐penetration bioimaging. Increasing the Mn^2+^ concentration causes the intensity of the VIS and NIR emission bands to increase up to their maximum values at *x* ≈ 0.025 and *x* ≈ 0.20, respectively, before monotonically decreasing because of concentration quenching. We use the pump‐power‐dependent UC emission (Figure S6, Supporting Information), the Stokes emission spectra (Figure S7, Supporting Information) and an analysis of the crystal structure to formulate a ground‐state absorption (GSA) and excited‐state absorption (ESA) UC mechanism for both the VIS and NIR emissions, which are shown in Figure 5b. In the GSA step, an electron of a pair (cluster) is excited from the $|{}^{2}F_{7/2}{,}^{6}A_{1}{(}^{6}S)〉\;(|{}^{2}F_{7/2}{,}^{6}A_{1}{(}^{6}S){}^{6}A_{1}{(}^{6}S)〉)$ ground state into the $|{}^{2}F_{5/2}{,}^{6}A_{1}{(}^{6}S)〉\;\left(|{}^{2}F_{5/2}{,}^{6}A_{1}{{(}^{6}S)}^{6}A_{1}{(}^{6}S)〉\right)$ intermediate excited state, which is mainly localized on the Yb^3+^ ion. In the ESA step, the electron is further promoted from the $|{}^{2}F_{5/2}{,}^{6}A_{1}{(}^{6}S)〉\;\left(|{}^{2}F_{5/2}{,}^{6}A_{1}{{(}^{6}S)}^{6}A_{1}{(}^{6}S)〉\right)$ state into the$|{}^{2}F_{7/2}{,}^{4}T_{1}{(}^{4}G)〉\;\left(|{}^{2}F_{7/2}{,}^{6}A_{1}{{(}^{6}S)}^{4}T_{1}{(}^{4}G)〉\right)$ emitting state, followed by the emission of a VIS (NIR) photon.

It is well known that the emission color of isolated Mn^2+^ ions can be tailored from green to deep red by changing the crystal field strength around the Mn^2+^ ion.^45^ Mn^2+^‐Mn^2+^ dimers are formed from two neighboring Mn^2+^ ions, and both the isolated Mn^2+^ ion and the Mn^2+^‐Mn^2+^ dimers have similar excitation spectra; thus, we speculated that the emission wavelength of the Mn^2+^‐Mn^2+^ dimers could also be tailorable by changing the host lattice (i.e., the ligand field strength). To investigate this hypothesis, further varieties of cubic perovskite AB_(1–*x*–*y*)_F_3_:Yb^3+^ *_y_*/Mn^2+^ *_x_* (*x* = 0.05, 0.20; *y* = 0.005; A = K, Rb, Cs; B = Mg, Zn, Cd) were synthesized (XRD patterns are shown in Figure S8, Supporting Information). UC emission is observed also from these Yb^3+^/Mn^2+^ codoped materials. At the lowest Mn^2+^ concentration, only a VIS UC emission band is found (Figure S9, Supporting Information), whereas NIR UC emission can be obtained at sufficiently high Mn^2+^‐doping concentration. Figure 5c shows the corresponding UC emission spectra. All spectra exhibit both VIS and NIR emission bands, with peaks of VIS UC emission at 605, 585, 567, 563, and 550 nm in KMgF_3_, KZnF_3_, KCdF_3_, RbCdF_3_, and CsCdF_3_, respectively. The corresponding NIR UC emission bands are centered at 765, 770, 795, 805, and 830 nm, respectively. These results show that the UC emission centers of the Yb^3+^‐Mn^2+^ pairs and the Yb^3+^‐Mn^2+^‐Mn^2+^ clusters have different intrinsic electronic structures in these Yb^3+^/Mn^2+^ doped fluorides. More importantly, both the VIS and the NIR emission bands have good tuning capabilities, which points to the great potential of this class of materials for various applications. In Figure 5d, the emission peak positions of the two emission bands are displayed as a function of the lattice constant *a*. As *a* increases, the VIS emission band monotonically blueshifts because of the decreasing crystal field strength (Figure 5e). However, the NIR emission band gradually redshifts with increasing *a*. Hence, and in accordance with our previous arguments, also here, the shift of the NIR emission band cannot be explained by considering isolated Mn^2+^ ions (Figure 5e).^43^, ^44^ Instead, the NIR emission band is generated by the ${}^{6}A_{1}{{(}^{6}S)}^{4}T_{1}{(}^{4}G)\to^{6}A_{1}{{(}^{6}S)}^{6}A_{1}{(}^{6}S)$ transitions of the coupled Mn^2+^‐Mn^2+^ entities, which could be a spin‐allowed (s = 1 to s = 1) transition^33^, ^46^ since the luminescence decay lifetime is shorter than 0.50 ms (Figure 2). In terms of excitation and emission spectra, and also in terms of the decay curve, the NIR emission behavior of ABF_3_:Yb^3+^/Mn^2+^ is similar to that of a d^8^‐configuration Ni^2+^ ion.^47^ Therefore, the ${}^{6}A_{1}{{(}^{6}S)}^{4}T_{1}{(}^{4}G)\to^{6}A_{1}{{(}^{6}S)}^{6}A_{1}{(}^{6}S)$ transitions of the coupled Mn^2+^‐Mn^2+^ dimers can be analogous to the ^3^ *T*_2_(^3^ *F*)→^3^ *A*_2_(^3^ *F*) transitions of Ni^2+^ ions. The Tanabe–Sugano diagram for the Ni^2+^ ion in an octahedral geometry shows that the spin‐allowed ^3^ *T*_2_(^3^ *F*)→^3^ *A*_2_(^3^ *F*) transitions can be finely tuned by changing the crystal field strength.^46^ In general, the higher of the crystal field strength, the shorter is the emission wavelength. This agrees well with the redshift of the NIR emission band in Yb^3+^/Mn^2+^ codoped ABF_3_ with increasing lattice constant (Figure 5c).

## 3 Discussion

In summary, we demonstrated tailoring of activator aggregation and super‐exchange in Mn^2+^‐Mn^2+^ effective dimers within the ABF_3_ fluoride perovskites. Such tailoring enables the promotion of distinct photoluminescence phenomena, denoted as anomalous photoluminescence, in the near‐infrared spectral range. In contrast to normal photoluminescence, we attribute anomalous photoluminescence from divalent manganese to the relaxation reaction of ${}^{6}A_{1}{{(}^{6}S)}^{4}T_{1}{(}^{4}G)\to^{6}A_{1}{{(}^{6}S)}^{6}A_{1}{(}^{6}S)$ that is facilitated by super‐exchange across Mn^2+^‐F‐Mn^2+^ bridges, i.e., effective dimers. This reaction occurs only when a sufficiently low interionic distance is obtained between Mn^2+^ species. We use a stochastic approach to estimate the crossover dopant concentrations for the occurrence of Mn^2+^ interaction within the first and second coordination shells. The results comply well with experimental observations of concentration quenching of photoluminescence from isolated Mn^2+^ (normal photoluminescence, quenched upon Mn^2+^—Mn^2+^ interaction) and from Mn^2+^—Mn^2+^ effective dimers (anomalous photoluminescence, quenched upon trimer formation). Tailoring of this procedure is achieved via adjusting the Mn—F—Mn angle and the Mn—F distance through substitution of the A^+^ and/or the B^2+^ species in the ABF_3_‐type compound. This affects the local ligand field strength (interionic distance) as well as the crystal symmetry (angular constraints) and enables tuning of the near‐infrared emission wavelength within 760–830 nm. DFT computational calculations and EXAFS analyses were performed to confirm this picture.

We applied this principle to produce pure anti‐Stokes near‐infrared emission at ≈760 nm from codoped KMgF_3_:Yb^3+^,Mn^2+^ upon excitation with a 976 nm laser diode, challenging the classical viewpoint where Mn^2+^ is used only for visible photoluminescence. In the present case, intense and tunable near‐infrared emission is generated. Such should be highly promising for future applications in biomedical imaging and labeling.

## 4 Experimental Section

*Materials Synthesis*: Perovskite fluorides of the type ABF_3_:Mn^2+^ (Mn^2+^/Yb^3+^) (A = Na^+^, K^+^, Rb^+^, and Cs^+^; B = Mg^2+^, Zn^2+^, Mn^2+^, and Cd^2+^) were synthesized through modified hydrothermal or solvothermal methods according to ref. 44. Further experimental details are provided in the Supporting Information.

*Characterization Methods*: Powder X‐ray diffraction data of the samples were recorded on an X‐ray powder diffractometer (Philips Model PW1830) with Cu Kα radiation (*λ* = 1.5406 Å) at a step width of 0.02° with a speed of 4° min^−1^. TEM images were carried out on a JEOL 2010 TEM operated at 200 kV. The room‐temperature (RT) excitation and emission spectra, and photoluminescence decay curves of the samples were recorded on an Edinburgh Instruments FLS920 spectrophotometer equipped with both continuous (450 W) and microsecond pulsed xenon (Xe) lamps as excitation sources. For temperature‐dependent emission and excitation spectra at 10–300 K, the samples were mounted on a cryostat (10–350 K, DE202, Advanced Research Systems). The UC emission spectra of the samples were measured with a Jobin–Yvon Triax 320 spectrofluorometer equipped with a R928 photomultiplier tube (PMT), in conjunction with a 976 nm diode laser as an excitation source. To characterize the thermal stability of UC PL at 300–473 K, the samples were placed on a TAP‐02 high‐temperature fluorescence instrument accessory (Tian Jin Orient‐KOJI Instrument Co., Ltd.). The X‐ray absorption fine structure (XAFS) data at Mn–K edge in both fluorescent and transmission mode were collected at the beamline BL14W1 in the Shanghai Synchrotron Radiation Facility (SSRF). The electron storage ring was operated at 3.5 GeV and 220 mA under a “top‐up” mode with a Si(111) double‐crystal monochromator for energy selecting. The photon energy was calibrated with the first inflection point of the Mn–K edge in a Mn metal foil. The raw XAFS data analysis was carried out using the IFEFFIT software package (Athena and Artemis).^48^

*Theoretical Calculations*: Geometry optimization for KMgF_3_:Mn^2+^ was performed using first‐principle calculations based on the DFT to investigate the possibility of Mn^2+^ aggregation in KMgF_3_. For this, the CASTEP (Cambridge Serial Total Energy Package, Accelrys, Inc.) code in Materials Studio was used.^49^ Details are provided in the Supporting Information.

## Supporting information

As a service to our authors and readers, this journal provides supporting information supplied by the authors. Such materials are peer reviewed and may be re‐organized for online delivery, but are not copy‐edited or typeset. Technical support issues arising from supporting information (other than missing files) should be addressed to the authors.

Supplementary
