# Supplementary material for: Tailored Near‐Infrared Photoemission in Fluoride Perovskites through Activator Aggregation and Super‐Exchange between Divalent Manganese Ions
Source: Adv Sci (Weinh). 2015 May 22;2(7):1500089. doi: 10.1002/advs.201500089 (PMC5115432; doi:10.1002/advs.201500089)
Supplement: Supplementary file 1 — Supplementary [file ADVS-2-0n-s001.pdf]

Copyright WILEY-VCH Verlag GmbH & Co. KGaA, 69469 Weinheim, Germany, 2015.

ADVANCED SCIENCE
Open Access

Supporting Information

for *Adv. Sci.*, DOI: 10.1002/advs.201500089

Tailored Near-Infrared Photoemission in Fluoride Perovskites through Activator Aggregation and Super-Exchange between Divalent Manganese Ions

*Enhai Song, Shi Ye, Tianhui Liu, Peipei Du, Rui Si, Xiping Jing, Sha Ding, Mingying Peng, Qinyuan Zhang, * and Lothar Wondraczek **

Copyright WILEY-VCH Verlag GmbH & Co. KGaA, 69469 Weinheim, Germany, 2013.

Supporting Information

Tailored near-infrared photoemission in fluoride perovskites through activator aggregation and super-exchange between divalent manganese ions

Enhai Song[1], Shi Ye[1], Tianhui Liu[2], Peipei Du[3], Rui Si[3], Xiping Jing[2], Sha Ding[1], Mingying Peng[1], Qinyuan Zhang[1,], and Lothar Wondraczek[4,]**

E. H. Song, Prof. S. Ye, S. Ding, Prof. M. Y. Peng, Prof. Q. Y. Zhang
State Key Laboratory of Luminescent Materials and Devices
Institute of Optical Communication Materials
South China University of Technology
Guangzhou, 510640, China
E-mail: qyzhang@scut.edu.cn

T. H. Liu, Prof. X. P. Jing
Beijing National Laboratory for Molecular Sciences, State Key Laboratory of Rare Earth Materials Chemistry and Applications, College of Chemistry and Molecular Engineering, Peking University, Beijing 100871, China

P. P. Du, Prof. R. Si
Shanghai Institute of Applied Physics, Chinese Academy Sciences, Shanghai Synchrotron Radiation Facility, Shanghai 201204, China

Prof. L. Wondraczek
Otto Schott Institute of Materials Research, University of Jena, 07743 Jena, Germany
E-mail: lothar.wondraczek@uni-jena.de

Supplementary Methods

Chemicals and materials. Reagents: Yb_2O_3 (99.998%), $C_4H_6MgO_4·4H_2O$ (99.98%), $C_4H_6MnO_4·4H_2O$ (99.99%), $C_4H_6ZnO_4·2H_2O$ (99.99%), $C_4H_6CdO_4·2H_2O$ (99.99%), KF (99.9%), NaF (99.9%), CsF (99.9%), RbF (99.9%), HNO_3 (analytical grade) and oleic acid (90%). Yb_2O_3 was supplied by Alfa Aesar Reagent Company, other chemicals were supplied by Aladdin Industrial Corporation. All of the chemicals were used as received without further purification.

Synthesis of $KMgF_3$:Yb^{3+}/Mn^{2+}. A series of $KMg_{(1\text{-}x)}F_3$: Mn^{2+}_x ($0.01 \leq x \leq 1.0$) and $KMg_{(1\text{-}x\text{-}y)}F_3$:$Yb^{3+}_y$/$Mn^{2+}_x$ ($0.01 \leq x \leq 0.40$; $y = 0.005$) samples were synthesised using a facile hydrothermal method. The typical synthesis procedures used in this study were as follows.

Firstly, a $Yb(NO_3)_3$ solution (0.02 mol/L) was obtained by dissolving Yb_2O_3 in hot HNO_3 solution. Then, 12 mmol of KF was completely dissolved in 10 mL of distilled water, and 15 mL of an aqueous solution containing stoichiometric amounts of $C_4H_6MgO_4 \cdot 4H_2O$, $C_4H_6MnO_4 \cdot 4H_2O$ and $Yb(NO_3)_3$ were dropped into the KF solution under vigorous stirring. After stirring for 20 minutes, the resulting transparent solution was transferred to a 50-mL Teflon-lined autoclave, which was held at 220 °C for 24 h. After reaction, the system was naturally cooled down to room temperature, and the products were collected and centrifuged several times with distilled water. Finally, absolute ethanol was used to remove any soluble residues, and the products were dried at 60 °C for 10 h.

Synthesis of $AMnF_3$ (A = K, Na and Cs). Here, 15 mL of ethanol and 5 mL of oleic acid were mixed under magnetic stirring to form a homogeneous solution. Then, 5 mL of an aqueous solution containing 4 mmol of $C_4H_6MnO_4 \cdot 4H_2O$ were added to the solution under vigorous stirring. Finally, 5 mL of an aqueous solution containing 12 mmol of KF (or NaF or CsF) were added to the complex, still stirring. The mixture was agitated for 30 min and transferred to a 50-mL autoclave. The autoclave was then sealed, and the mixture hydrothermally treated at 220 °C for 24 h. After the reaction, the system was naturally cooled back to room temperature. The obtained product was centrifuged, washed and dried as above.

Synthesis of $AB_{(1\text{-}x\text{-}y)}F_3$:$Yb^{3+}y$/$Mn^{2+}x$ ($x = 0.05$ and 0.20; $y = 0.005$)(A = K, Rb and Cs; B = Mg, Zn and Cd). Again, 15 mL of ethanol and 5 mL of oleic acid were mixed and magnetically stirred to form a homogeneous solution. Then, 5 mL of an aqueous solution containing stoichiometric amounts of $C_4H_6MgO_4 \cdot 4H_2O$, $C_4H_6MnO_4 \cdot 4H_2O$ and $Yb(NO_3)_3$ were added to the solution under vigorous stirring. Finally, 5 mL of an aqueous solution containing 12 mmol of KF was added to the complex. The mixture was agitated for 30 min and transferred to a 50-mL autoclave. The autoclave was then sealed,

and the mixture hydrothermally treated at 220 °C for 24 h. After the reaction was completed, the system was naturally cooled down to room temperature The obtained product was centrifuged, washed and dried as above.

Theoretical calculations. In the CASTEP code, the wave functions of valence electrons were expanded in an ultrasoft pseudopotential plane-wave basis with the specific cut-off energy E_{cut}. The exchange-correlation functional employed was the Perdew, Burke and Ernzerhof (PBE) implementation of the spin-polarized Generalized Gradient Approximation (GGA).[1,2] It is reported that the GGA would result in more exact data than the Local Density Approximation (LDA) for spin polarized system.[1-3] Six different 2×2×4 supercells (80 atoms) with two Mg^{2+} ions substituted by two Mn^{2+} ions according to the ICSD card No. 94089 structure model were used as initial models. The initial supercell parameters were a = b = 7.9794 Å, c = 15.9588 Å. Each geometry optimization by Broyden-Fletcher-Goldfarb -Shannon (BFGS) alogrithm[4] was performed to the model. Both E_{cut} and k-point mesh were tested and were set to 310 eV and a 3×3×3 Monkhorst-Pack grid, which were sufficient for energy convergence. The structures were considered to be converged when the force on each ion was less than ~0.05 eV/Å, with a maximum displacement of ~0.002 Å and a convergence in the total energy of about 2×10^{-6} eV/atom. To understand the coupling interaction between Mn^{2+} ions, the electronic spin alignment for Mn^{2+} ions in the $KMgF_3$ host were set to be FM and AFM. That is, possible FM coupling and AFM coupling for Mn^{2+} aggregates were adopted in the geometry optimization. The energy difference of the two coupling modes for each model were also calculated ($\Delta E = E_{\mathrm{AFM}} - E_{\mathrm{FM}}$).

Supplementary Tables and Table captions:

Table S1. Initial lattice constants for a 2×2×4 supercell of $KMgF_3$.

Lattice constants	value
a (Å)	7.9794
b (Å)	7.9794
c (Å)	15.9588
α (degree)	90.0000
β (degree)	90.0000
γ (degree)	90.0000
V (Å^3)	1016.1100

Table S2. Optimized cell parameters and forming energy (*E*) of six possible substitution geometry modes (denoted as M1, M2, M3, M4, M5 and M6) for a 2×2×4 supercell of $KMgF_3$:Mn^{2+} with two Mg^{2+} ions substituted by two antiferromagnetic coupling (AFM) Mn^{2+} ions.

Parameters	M1	M2	M3	M4	M5	M6
a (Å)	8.2874	8.2865	8.2867	8.2901	8.2853	8.2856
b (Å)	8.2874	8.286548	8.284929	8.290169	8.2853	8.2856
c (Å)	16.5722	16.5661	16.5629	16.5561	16.5676	16.5563
α (degree)	89.9999	89.9999	90.0000	90.0000	90.0000	90.0000
β (degree)	90.0000	90.0000	90.0000	90.0000	90.0002	89.9999
γ (degree)	89.9999	90.0000	89.9999	89.9999	89.9998	90.0000
V (Å^3)	1138.2076	1137.5432	1137.1298	1137.8466	1137.3173	1136.6289
E_{AFM} (eV/mol)	-59330.9750	-59330.9671	-59330.9896	-59330.9701	-59330.9837	-59330.9744
ΔE_a (eV/mol)	0.0000	0.0079	-0.0146	0.0049	-0.0087	0.0006
Two shortest Mn-	4.1437	8.2865	5.8596	9.2686	7.1755	10.1474
Mn distances (Å)	8.2874	8.2865	8.2867	8.2901	8.2853	8.2856

$\Delta E_a = E_{AFM}(\mathrm{Mi}) - E_{AFM}(\mathrm{M1})$ (i = 1, 2, 3, 4, 5 and 6).

Table S3. Optimized cell parameters and forming energy (E) of six possible substitution geometry modes (denoted as M1, M2, M3, M4, M5 and M6) for a 2×2×4 supercell of $KMgF_3:Mn^{2+}$ with two Mg^{2+} ions substituted by two ferromagnetic coupling (FM) Mn^{2+} ions.

Parameters	M1	M2	M3	M4	M5	M6
a (Å)	8.2892	8.2867	8.2873	8.2895	8.2864	8.2903
b (Å)	8.2892	8.28678	8.2859	8.2912	8.2864	8.2903
c (Å)	16.5712	16.5677	16.5625	16.5582	16.5656	16.5557
α (degree)	90.0000	90.0000	90.0000	90.0000	89.9999	90.0000
β (degree)	90.0000	90.0000	90.0000	90.0000	90.0000	90.0000
γ (degree)	90.0000	90.0000	89.9999	90.0000	90.0000	90.0000
V (Å^3)	1138.6469	1137.72135	1137.3273	1137.7748	1137.4788	1138.8743
E_{FM} (eV/mol)	-59330.9353	-59330.9644	-59330.9888	-59330.9709	-59330.9833	-59330.9649
ΔE_b (eV/mol)	0.0000	-0.0291	-0.0535	-0.0356	-0.0480	-0.0296
Two shortest Mn-	4.1446	8.2867	5.8573	9.2585	7.1752	10.1433
Mn distances (Å)	8.2892	8.2867	8.2873	8.2895	8.2864	8.2903

$\Delta E_b = E_{FM}(Mi) - E_{FM}(M1)$ (i = 1, 2, 3, 4, 5 and 6).

Table S4. Forming energy difference between E_{AFM} and E_{FM} of the six possible substitution geometry modes (denoted as M1, M2, M3, M4, M5 and M6) for a 2×2×4 supercell of $KMgF_3$:Mn^{2+} with two Mg^{2+} ions substituted by two Mn^{2+} ions

Parameters	M1	M2	M3	M4	M5	M6
E_{AFM} (eV/mol)	-59330.9750	-59330.9671	-59330.9896	-59330.9701	-59330.9837	-59330.9744
E_{FM} (eV/mol)	-59330.9353	-59330.9644	-59330.9888	-59330.9709	-59330.9833	-59330.9649
ΔE (eV/mol)	-0.0397	-0.0027	-0.0008	0.0008	-0.0004	-0.0095

$\Delta E = E_{AFM} - E_{FM}$.

Supplementary Figures and Figure captions:

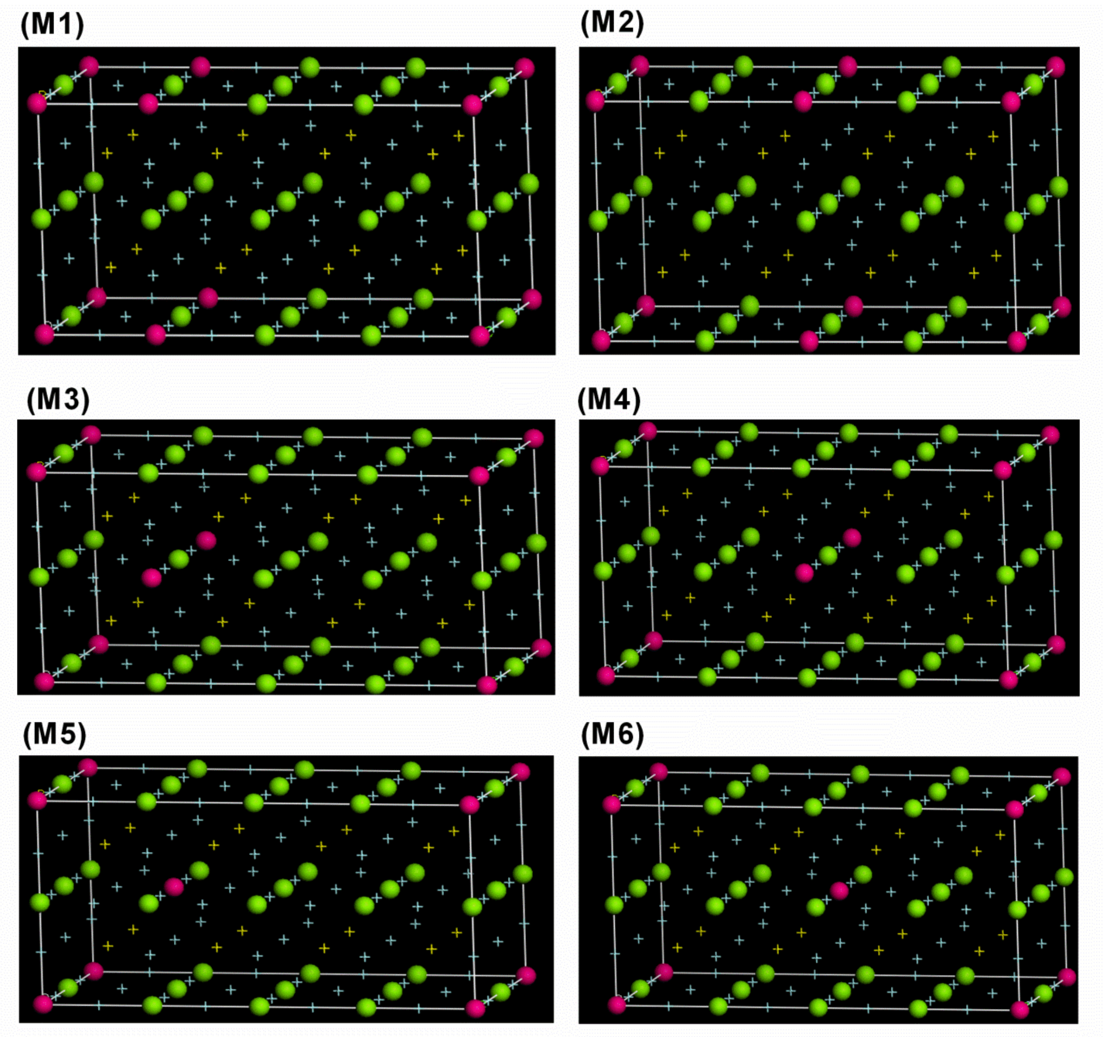

Figure S1. Six possible substitution geometry models (denoted as M1, M2, M3, M4, M5 and M6) for a 2×2×4 supercell of $KMgF_3$:Mn^{2+} with two Mg^{2+} ions substituted by two Mn^{2+} ions. The green ball, red ball, indigo + and yellow + denote as Mg, Mn, F and K ions, respectively.

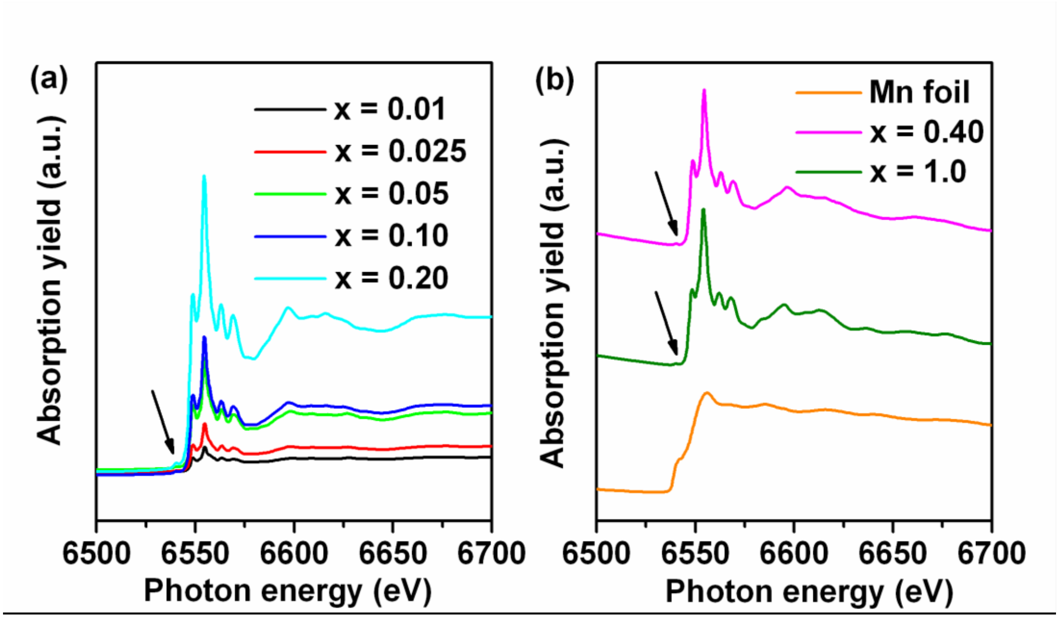

Figure S2. (a) XANES spectra of $KMg_{(1-x)}F_3:Mn^{2+}_x$ (0.01≤ x ≤0.20) (fluorescence mode). **(b)** XANES spectra of $KMg_{(1-x)}F_3:Mn^{2+}_x$ (x = 0.40 and 1.0) and Mn foil (transmission mode). The spectra of $KMg_{(1-x)}F_3:Mn^{2+}_x$ (0.01≤ x ≤1.0) are mutually quite similar, but distinctly different from the reference spectrum of the Mn foil, confirming the presence of Mn^{2+} dopants in $KMgF_3$ host, rather than secondary phases. For all Mn^{2+}-containing perovskite samples, the characteristic peak in the first derivative of the edge spectra was observed at ~6549 eV, and a very weak pre-edge feature was consistently observed at ~6539 eV (**arrow in Fig. S2**), indicating the valence state of Mn in this system is +2.

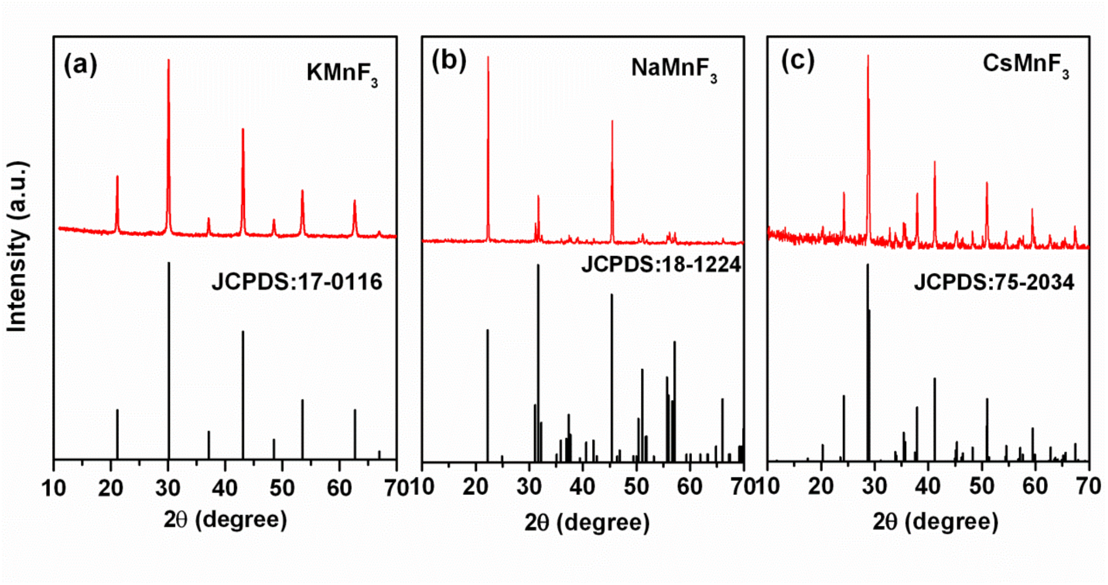

Figure S3. X-ray powder diffraction patterns of as-recieved $KMnF_3$, $NaMnF_3$ and $CsMnF_3$ nanocrystals. Joint Committee on Powder Diffraction Standards (JCPDS) files number 17-0116, 18-1224 and 75-2034 are included for comparison. The compound $KMnF_3$ exhibits a typical cubic perovskite structure with Pm-3m space group (No. 221) and the lattice constant *a* = 4.189 Å. The compound $NaMnF_3$ is an orthorhombic perovskite with Pnma space group (No. 62) and lattice constants *a* = 5.748 Å, *b* = 8.004 Å and *c* = 5.551 Å. The compound $CsMnF_3$ is a hexagonal perovskite structure with P63/mmc space group (No. 194) and lattice constants *a* = *b* = 6.225 Å and *c* = 5.551 Å.

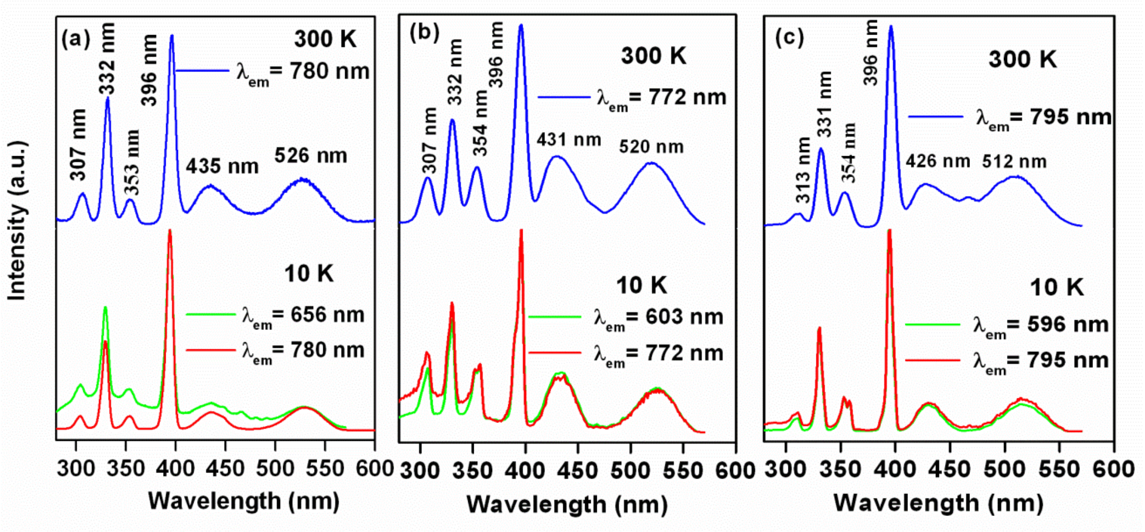

Figure S4. Excitation spectra of samples $KMnF_3$ (a), $NaMnF_3$ (b) and $CsMnF_3$ (c) at 10 and 300 K. The six typical excitation peaks between 300 and 570 nm can be assigned to the electronic transitions of Mn^{2+} from the ground state $^6A_1(S)$ to the excited states $^4T_1(P)$, $^4E(D)$, $^4T_2(D)$, $[^4A_1(G), {}^4E(G)]$, $^4T_2(G)$ and $^4T_1(G)$.

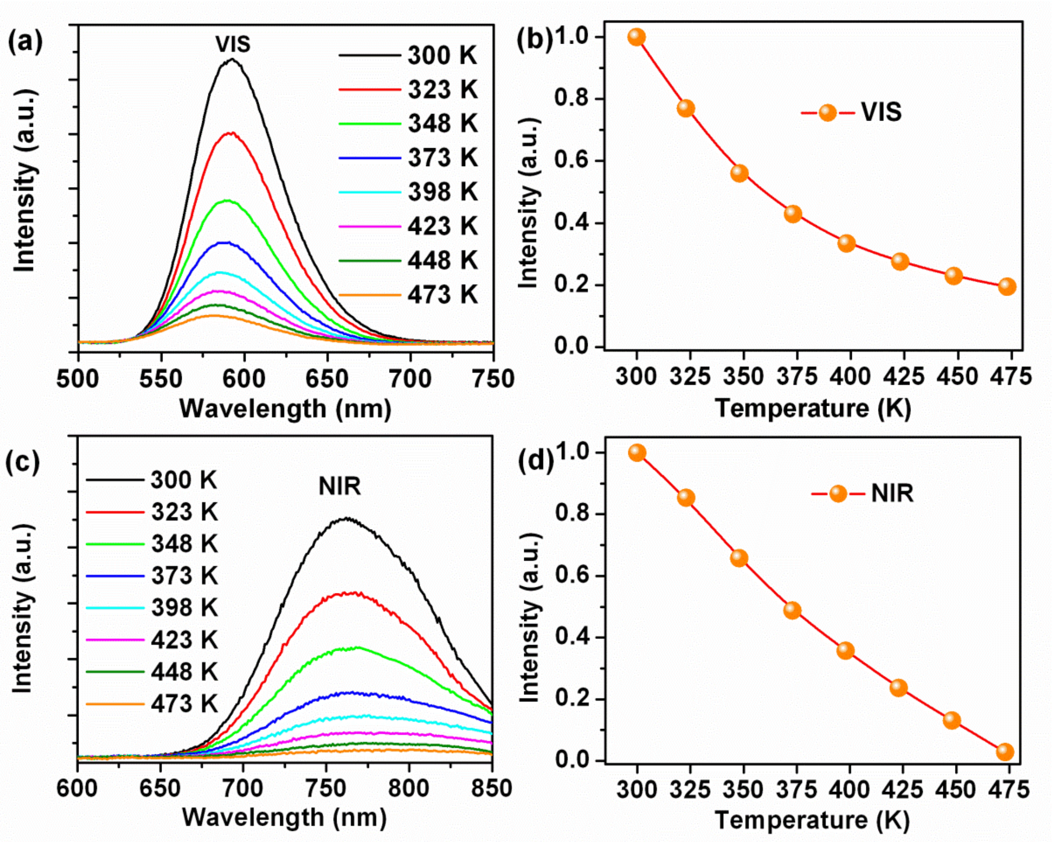

Figure S5. Temperature-dependent UC emission spectra and integrated intensity of $KMg_{(1-x-y)}F_3:Yb^{3+}{}_y,Mn^{2+}{}_x$ (x = 0.01; y = 0.005) (**a** and **b**) and $KMg_{(1-x-y)}F_3:Yb^{3+}{}_y,Mn^{2+}{}_x$ (x = 0.20; y = 0.005) upon excitation with a 976- nm LD at a power density of 10 Wcm^{-2}.

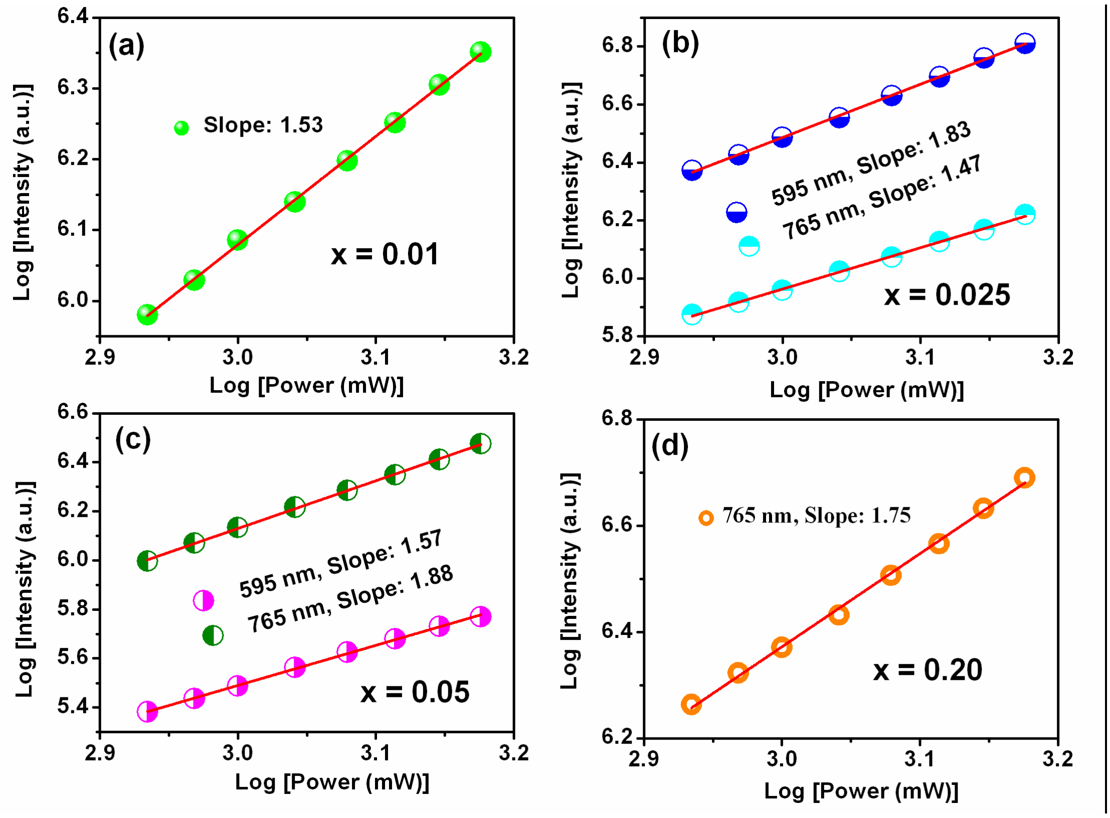

Figure S6. Plots of the upconversion emission intensity versus 976 nm laser pumping power for the two emission bands (VIS/NIR) of Mn^{2+} in $KMg_{(1-x-y)}F_3:Yb^{3+}{}_y,Mn^{2+}{}_x$ ($0.01 \leq x \leq 0.20$; $y = 0.005$). (**a**) ($x = 0.01$; $y = 0.005$), (**b**) ($x = 0.025$; $y = 0.005$), (**c**) ($x = 0.05$; $y = 0.005$), and (**d**) ($x = 0.20$; $y = 0.005$).

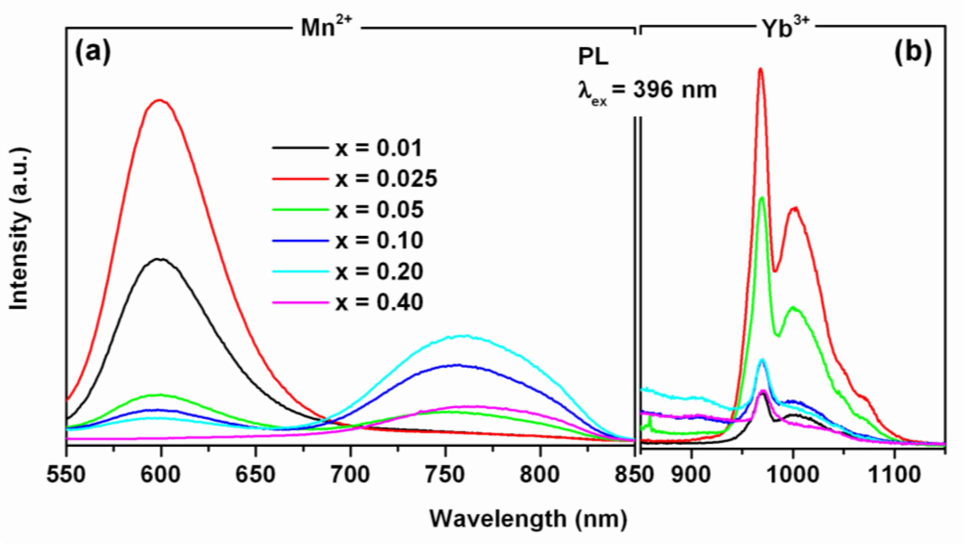

Figure S7. Room-temperature Stokes emission spectra of $KMg_{(1\text{-}x\text{-}y)}F_3$:$Yb^{3+}{}_y$,$Mn^{2+}{}_x$ ($0.01 \leq x \leq 0.40$; $y = 0.005$) upon 396 nm light excitation. (**a**) Mn^{2+}-emission in the range of 550-850 nm and Yb^{3+}-emission in the range of 850-1200 nm (**b**). It can be observed that the Stokes emission spectra of $KMg_{(1\text{-}x\text{-}y)}F_3$:$Yb^{3+}{}_y$,$Mn^{2+}{}_x$ ($0.01 \leq x \leq 0.40$; $y = 0.005$) are similar to those of $KMg_{(1\text{-}x)}F_3$: $Mn^{2+}{}_x$ ($0.01 \leq x \leq 0.40$) (Fig.2a) upon 396 nm light excitation. The bands centered at 595 and 760 nm can be ascribed to the $^4T_1(^4G)\rightarrow{}^6A_1(^6S)$ transitions of isolated Mn^{2+} ions and $^6A_1(^6S)^4T_1(^4G)\rightarrow{}^6A_1(^6S)^6A_1(^6S)$ transitions of Mn^{2+}-Mn^{2+} dimers, respectively. Additionally, the Yb^{3+}-emission centered at 972 nm ($^2F_{5/2}\rightarrow{}^2F_{7/2}$) is also observed in Yb^{3+}-Mn^{2+} codoped $KMgF_3$ samples through Mn^{2+} excitation, indicating that Mn^{2+} and Yb^{3+} formed some unique emission centers in this system since Mn^{2+} and Yb^{3+} ions have no spectral overlap.

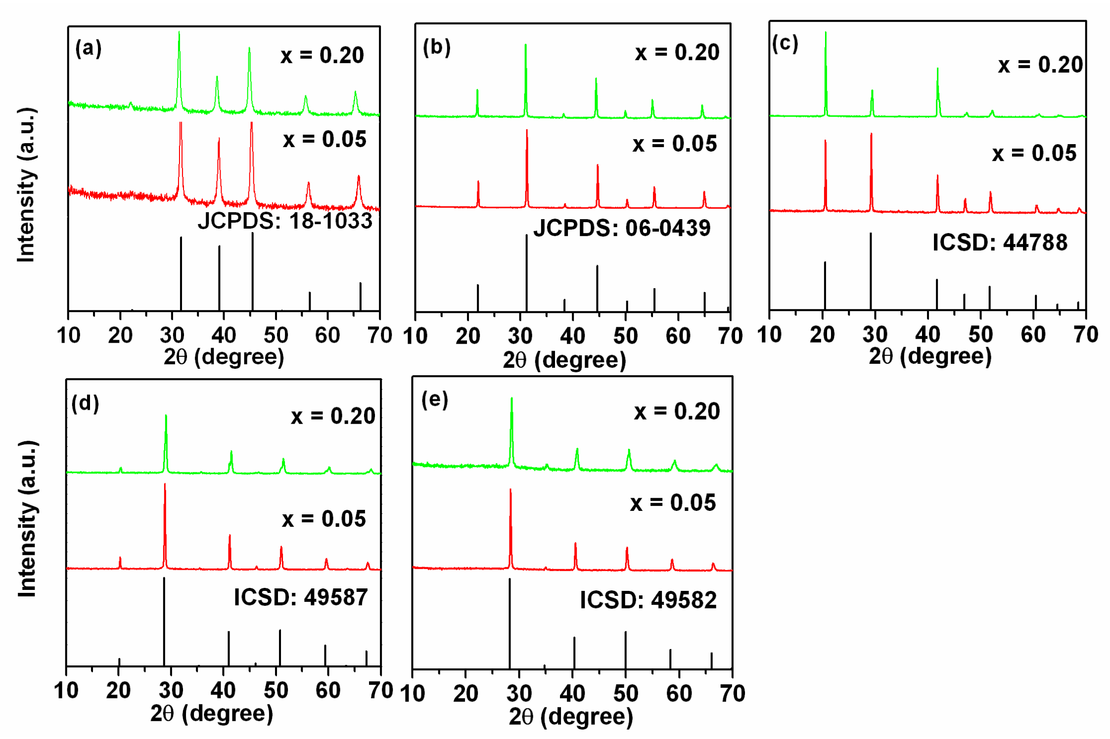

Figure S8. X-ray powder diffraction patterns of $AB_{(1-x-y)}F_3$:$Yb^{3+}{}_y$/$Mn^{2+}{}_x$ (x = 0.05 and 0.20; y = 0.005) (A = K, Rb and Cs; B = Mg, Zn and Cd) and corresponding reference data (JCPDS or inorganic crystal structure database, ICSD). Yb^{3+}/Mn^{2+} codoped (a) $KMgF_3$, (b) $KZnF_3$, (c) $KCdF_3$, (c) $RbCdF_3$ and (c) $CsCdF_3$.

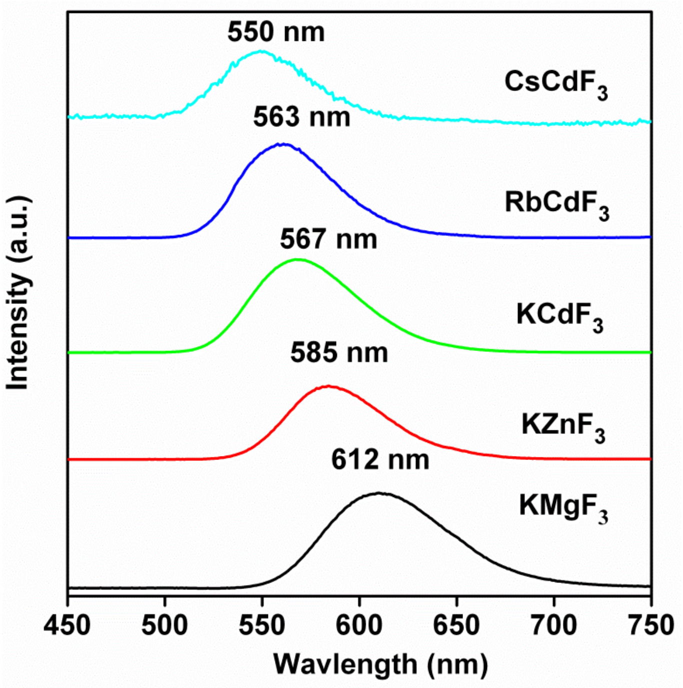

Figure S9. Room-temperature upconversion emission spectra of $AB_{(1-x-y)}F_3$:$Yb^{3+}{}_y$/$Mn^{2+}{}_x$ (x = 0.05, y = 0.005) (A = K, Rb and Cs; B = Mg, Zn and Cd) upon excitation with a 976-nm laser diode at a power density of 10 Wcm^{-2}.

Supplementary References

[1] J. P. Perdew, K. Burke, M. Ernzerhof, *Phys. Rev. Lett.* **1996**, *77*, 3865.

[2] J. P. Perdew, Y. Wang, *Phys. Rev. B* **1992**, *45*, 13244.

[3] M. A. Kamran, R. B. Liu, L. J. Shi, Z. A. Li, T. Marzi, C. Schöppner, M. Farle, B. S. Zou, *Nanotech.* **2014**, *25*, 385201.

[4] B. G. Pfrommer, M. Côté, S. G. Louie, M. L. Cohen, *J. Comput. Phys.* **1997**, *131*, 233.
